# Association between social skills and mental health outcomes in Health Science Residents in Brazil

**DOI:** 10.1371/journal.pone.0341773

**Published:** 2026-02-18

**Authors:** Carina Rodrigues da Silva, Andreia Gonçalves Arruda, João Wellinton Pletti, Ana Amélia Domingues Gomes, Thaís Rabelo dos Santos-Doni, Rafael Felipe da Costa Vieira, Alexandre Coutinho Antonelli, Dagma Venturini Marques Abramides, Alexandre Redson Soares da Silva

**Affiliations:** 1 Graduate Program in Veterinary Sciences in the Semi-arid Region, Agricultural Sciences Campus, Federal University of São Francisco Valley, Petrolina, PE, Brazil; 2 Department of Veterinary Preventive Medicine, College of Veterinary Medicine, Ohio State University, Columbus, Ohio, United States of America; 3 Faculty of Dentistry of Bauru, University of São Paulo, Bauru, SP, Brazil; 4 Institute of Agricultural Sciences, Unaí Campus, Federal University of the Jequitinhonha and Mucuri Valleys, Unai, MG, Brazil; 5 Center for Computational Intelligence to Predict Health and Environmental Risks, University of North Carolina at Charlotte, Charlotte, North Carolina, United States of America; 6 Department of Epidemiology and Community Health, University of North Carolina at Charlotte, Charlotte, North Carolina, United States of America; University of Missouri School of Medicine, UNITED STATES OF AMERICA

## Abstract

Residency programs play a crucial role in the development and technical training of young health professionals. However, mental health issues such as psychological distress, anxiety, and depression are prevalent, often influenced by work satisfaction and the learning environment. Despite their relevance, studies investigating the prevalence and associated factors of these conditions across medical, multidisciplinary, and veterinary residency programs remain scarce. This study aimed to determine the prevalence of psychological distress, anxiety, and depression, and to examine their association with social skills among residents in Brazilian health science programs. We conducted a voluntary online survey with residents from three major residency programs (medical, multidisciplinary, and veterinary) across different Brazilian institutions. The cross-sectional study sample consisted of a total of 51 participants. Mental health outcomes were assessed using validated instruments: the Resident Questionnaire (RQ) for emotional distress, General Anxiety Disorder (GAD-7) for anxiety, and Patient Health Questionnaire (PHQ-9) for depression. The Multidimensional Scale of Social Expression (MSSE-M) measured social skills. Multivariable mixed-effects linear regression models addressed each mental health outcome separately, with social skill scores as primary explanatory variables. The results revealed a high prevalence of emotional distress (86.3%), anxiety (58.8%), and depression (56.9%) among residents. Greater satisfaction with the learning environment was significantly associated with lower mental health symptom levels. Residents demonstrated a generally satisfactory overall repertoire of social skills (60.78%), with the “Start and keeping conversations” and “Express positive affect” subscales scoring highest across all programs. For emotional distress, the “ability to defend rights” subscale was significantly associated with its reduction (−0.58), while a broader overall social skill repertoire was significantly associated with reductions in anxiety (−3.14) and depression (−3.92). These findings highlight the significant burden of mental health challenges among residents and emphasize the role of a supportive environment. Social skills training may help mitigate psychological distress and support resident well-being.

## Introduction

The professional residency period in the health sciences field is essential for professional growth, as it integrates recent graduates into the work environment, allowing specialization and improvement in their chosen area of work through theoretical and practical experiences [1]. This phase of professional and personal training is crucial for preparing professionals to face adversities, motivating young professionals to immerse themselves in postgraduate training work in search of solidifying theoretical and practical knowledge that complements their undergraduate education [2].

Residency is considered a challenging period due to intense demands for excellence from patients and supervisors, high levels of stress in the work environment and interpersonal relationships, work overload, and accumulation of academic activities [3–5]. Because of that, during this period, many residents experience a significant increase in mental health issues such as psychological distress, anxiety, and depression [5–8]. This trend is internationally supported, with studies consistently showing a substantial mental health burden among medical residents globally [9]. In veterinary medicine, recent evidence highlights concerning levels of mental health problems among interns and residents, with elevated rates of depression and suicidal ideation compared to the general population [10,11]. Additionally, factors such as being female [8,12], experiencing stressful events [6,8], and having low subjective well-being [13] have been associated with depression during residency training. These mental health issues can lead to severe consequences, including increased risk of suicidal thoughts, occurrence of medical errors, and reduced adherence to safety and practice standards [8,14–16]. Such consequences not only affect the well-being of residents but may also directly impact the patients and clients under their care [14–16].

Beyond individual-level factors, the residency program’s environment itself can significantly affect the mental health of residents [5]. Previous studies have linked the clinical learning environment within residency programs to the quality of education, performance, and overall well-being of residents [2,17,18]. Furthermore, studies showed that this professional environment is associated with resident depression, particularly when factors such as high effort, low reward, and lack of job autonomy are present [19–21]. Similar concerns extend beyond medicine, as researchers described veterinary and other health science residency programs as highly demanding, with limited institutional support to mitigate the psychological burden on trainees [10,11,22].

For these professionals, technical inexperience and lack of social skills can contribute to emotional exhaustion, psychological distress, sleep deprivation, psychoactive substance abuse, and work/life imbalance [5,23]. Given that residents commonly begin their practice in an unfamiliar context, it is essential for them to have an adequate repertoire of technical and social skills, as these are equally important in both professional and personal settings. Such abilities include communication, expressing positive and negative feelings, as well as civility, assertiveness, and empathy [24]. Social skills also play a key role in supporting resilience and well-being in clinical practice, fostering better teamwork and reducing burnout risk, as suggested by recent studies in interprofessional health education and veterinary training [10,22,25]. Given their relevance to health practices, several studies have focused on the psychosocial characteristics of professionals [3,7,26–28].

Despite the documented importance of social skills in improving professionals’ resilience, there is a lack of studies directly investigating their association with mental health among physicians, multidisciplinary, and veterinary professionals. The aim of our study was to determine the prevalence of mental health issues, including emotional distress, anxiety, and depression, amongst medical, multidisciplinary, and veterinary residents; to investigate the association between the repertoire of social skills and these mental health issues, while accounting for other important factors, such as satisfaction with the work and learning environment. We hypothesized that higher levels of social skills would be inversely associated with symptoms of anxiety, depression, and emotional distress.

## Materials and methods

Ethics Committee approval was obtained before data collection from the Ethics Committee for Research with Human Beings of the Faculty of Dentistry of Bauru of the University of São Paulo (protocol code 02778218.3.0000.5417 and date of approval 15 February 2019); and the Federal University of São Francisco Valley (protocol code 02645518.6.0000.5196 and date of approval 11 July 2019) and registered with the Brazilian National Research Ethics Commission (CONEP), under the Ministry of Health. We obtained informed consent from all subjects involved in the study.

### Study design, population and data collection procedures

This cross-sectional study employed an online survey instrument for data collection. The study population comprised medical, multidisciplinary, and veterinary residents enrolled in their respective residency programs across five institutions in Brazil. For the purposes of this study, residents were defined as professionals enrolled in Brazilian residency programs accredited by the Ministries of Health and Education. These programs are classified into three distinct modalities: medical, multidisciplinary, and veterinary.

The Multidisciplinary Residency Program in Brazil is a *lato sensu* postgraduate course structured as in-service training conducted within hospital units and affiliated health networks. Its primary objective is to integrate qualified professionals into priority areas of *Sistema Único de Saúde –* Brazilian Unified Health System (SUS), encompassing various health professions. These include, but are not limited to, veterinary medicine, biomedicine, biological sciences, physical education, nursing, pharmacy, physiotherapy, speech therapy, nutrition, dentistry, psychology, social work, and occupational therapy (Resolution CNS nº 287/1998; [27,28]). This program typically has a duration of approximately two years, with a minimum workload of 6,000 hours, and operates on a full-time, exclusive dedication basis, usually financed by a scholarship [29,30].

The Medical Residency Program also shares characteristics of a *lato sensu* postgraduate course in the form of in-service training, but it is exclusively directed towards physicians. It aims to specialize doctors in specific medical areas. Medical residency programs can be direct access, allowing physicians to enter without a prior specialty, or require a prerequisite, where completion of another residency is necessary. The weekly workload is 60 hours, including theoretical and practical activities, with a duration varying from 1 to 5 years depending on the specialty.

While legislatively often categorized under the multidisciplinary modality, in this study, the veterinary residency was considered as a separate category due to its specific and exclusive focus on animal medicine and the inherent particularities of veterinary training. This modality seeks technical-scientific specialization in specific areas of veterinary medicine, with a workload and duration comparable to other residency programs.

The health residency programs, established by Law No. 11,129 of 2005, are guided by the principles and guidelines of the SUS, based on local and regional needs and realities. Within these programs, the requirement for ‘shifts’ varies according to the specialty’s nature. In this context, shifts are defined as periods of continuous, supervised clinical activity in urgent, emergency, or 24-hour units, limited to a maximum of 24 uninterrupted hours, including overnight and/or weekend rotations. While some curricula demand intensive practical scales, others may not require any shifts, such as modalities focused on laboratory, diagnostic, or public health areas that may fulfill their requirements through outpatient or administrative schedules. This classification allows for a better understanding and analysis of the profiles and needs of residents in different professional contexts, contributing to the planning of educational and health policies aimed at these groups.

The five institutions that gave rise to the study subjects (source population) were conveniently selected based on co-author’s professional networks with the residency program coordinators. Four of them were located in the states of São Paulo and one in the state of Pernambuco. The institutions selected for the study had their specific names hidden to protect confidentiality of participants, but they all needed to be accredited by the Brazilian Ministries of Health and Education to be included in the study.

To recruit individual participants, the residency coordinators for each institution collaborated with each university’s Human Resources department, which compiled a comprehensive list of residents enrolled in the participating institutions. Based on this list provided, recruitment was conducted through email and social media platforms (e.g., WhatsApp^©^), with invitations sent either by the research team, institutional administrative staff, or resident representatives, depending on the institution’s internal protocols. Participation was voluntary, with no incentives offered for participation in the study. All active residents were eligible to participate in the study, regardless of the length of time enrolled in their respective programs.

**Fig 1:**
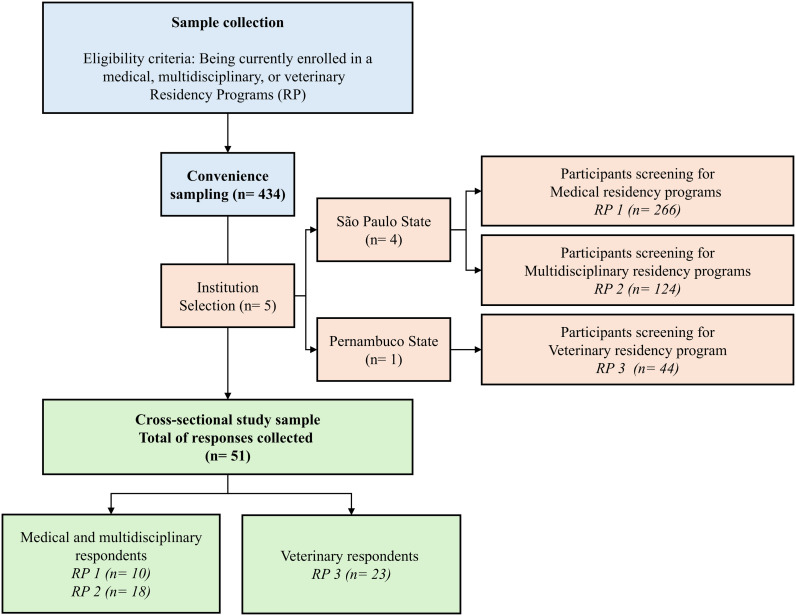
Flowchart of study design and sample collection.

Weekly recruitment efforts were conducted, and the questionnaire was available for responses from August 6, 2019, to November 11, 2019. Participation was confirmed by study subjects through online agreement after a description of the study objectives and agreement to the Free and Informed Consent Form, which was approved by Ethics Committee for Research with Human Beings of the Faculty of Dentistry of Bauru at the University of São Paulo (protocol code 02778218.3.0000.5417); and the Federal University of São Francisco Valley (protocol code 02645518.6.0000.5196). None of the institutions mentioned were included in the study. Participant’s answers were collected anonymously. The source population comprised 434 residents enrolled in medical (n = 266), multidisciplinary (n = 124), and veterinary (n = 44) residency programs. Of the 434 eligible residents, 51 completed the survey, yielding a response rate of 11.75%. The final study sample included 10 residents from medical programs, 18 from multidisciplinary programs, and 23 from veterinary programs. A flowchart detailing the study design and participant selection process is provided in Fig 1, which includes the distribution of participants across the institutions (Medical and Multidisciplinary residents from four institutions in São Paulo, and veterinary residents from one institution in Pernambuco).

### Questionnaire development

The questionnaire, developed using Google Forms^©^, comprised six sections (instruments) and had an estimated average completion time of 40 minutes. Before data collection, it was internally tested by approximately five members of the study team, and feedback led to minor adjustments. Participants responded asynchronously, beginning with questions related to sociodemographic data and professional activities. The survey was completed anonymously, and no personally identifying data was collected or disclosed, ensuring full compliance with the Brazilian General Data Protection Law (LGPD) – Law 13,709/2018. They then proceeded to the validated instruments: Resident Questionnaire (RQ); General Anxiety Disorder-7 (GAD-7); Patient Health Questionnaire-9 (PHQ-9); and Multidimensional Scale of Social Expression – Motor Subscale (MSSE-M). The interpretation of all validated instruments and their score calculations are detailed in Table 1.

**Table 1:** Detailing of questionnaire’s validated instruments subscales, internal reliability, items numbers, and scoring interpretation.

Questionnaire	Cronbach’s α	Number of items	Scoring
**Resident Questionnaire – RQ**	**0.89**	28	Likert scale (1–5)
Emotional distress	0.89	11	Lower scores indicate reduced levels of emotional distress
Workload satisfaction	0.74	8	Higher scores indicate greater satisfaction with workload
Learning environment satisfaction	0.76	9	Higher scores indicate greater satisfaction with the learning environment
**General Anxiety Disorder – GAD7**	**0.91**	7	Likert scale (0–3) Positive indicators of anxiety: scores greater than 10; Absence: scores below 5; Mild: 5–9; Moderate: 10–14; Severe: 15–21
**Patient Health Questionnaire - PHQ9**	**0.84**	9	Likert scale (0–3) None-minimal: 0–4; Mild: 5–9; Moderate: 10–14; Moderately Severe: 15–19; Severe: 20–27
**Multidimensional Scale of Social Expression – Motor subscale – MSSE-M**	**0.87**	46	Likert scale (0–4) Higher scores reflect a broader and more effective repertoire of social skills. Scores above the study sample’s mean indicate satisfactory social competence
Start and keeping conversations	0.81	11	
Making refusals	0.37	3	
Acknowledge compliments	0.53	3	
Speaking in public	0.68	3	
Expressing positive affect	0.72	9	
Expressing negative affect	0.62	7	
Expressing disagreement	0.63	5	
Defending rights	0.58	5	

The first part of the survey, “Socio-demographic and professional activities”, included 20 questions on demographic and work-related information including sex, age, marital status, number of children, type and current year of the residency program, number of shifts by month, and weekly duty hours. The full questionnaire is included in Supporting Information File (S1 File).

To obtain information regarding residents’ perspectives on their programs and mental health, three components were measured using a 28-item Resident Questionnaire (RQ): workload satisfaction scale, learning environment satisfaction scale and emotional distress scale [31,32]. The workload satisfaction scale included 8 items such as call schedule, caseload, excess workload, allocated time for reading, clerical and administrative support, hospital support services, time demands, and workups. The learning environment satisfaction scale comprised 9 items concerning faculty feedback, counseling and support, learning experiences during in-patient rotations and scheduled conferences, instructions received, and cooperation among residents. The emotional distress scale included 11 items addressing feelings of overwhelm anxiety, isolation, self-doubt, and challenges with work-life balance. For all these scales participants provided their level of agreement with each statement on a five-point Likert scale, ranging from “strongly disagree” to “strongly agree”. The interpretation of the questionnaire was, for the work-related components, the higher the scores, the higher satisfaction with the work environment, and for emotional distress, the lower the scores the lower the stress level.

Anxiety was measured using the General Anxiety Disorder 7 (GAD-7), an instrument developed for diagnosing and monitoring the occurrence of anxiety symptoms in respondent’s last two weeks [33]. It is composed of seven items, arranged on a four-point Likert scale, from 0 (never) to 3 (almost every day). Punctuation greater than 10 is interpreted as indicators of anxiety. The instrument also evaluates the severity of anxiety symptoms considering scores below 5 to be the absence of anxiety, mild from five to nine, moderate from 10 to 14, and severe from 15 to 21.

We used the Patient Health Questionnaire (PHQ-9) to measure depression. It is an instrument consisting of nine questions that seek to detect symptoms that indicate a major depressive episode in the general population [34,35]. The nine symptoms consist of depressed mood, anhedonia (loss of interest or pleasure in doing things), problems with sleeping, tiredness, or lack of energy, change in appetite or weight, feelings of guilt or worthlessness, trouble concentrating, feeling sluggish or restless and suicidal thoughts. We evaluated the frequency of each symptom in the last two weeks on a Likert scale from 0 (never) to 3 (almost every day). The PHQ-9 scores were calculated adding the values of each participant response on the Likert scale. The absence or minimal depressive symptoms had scores ranging from 0 to 4; mild from 5 to 9; moderate from 10 to 14; moderately severe from 15 to 19 and severe from 20 to 27.

We assessed social skills using the Multidimensional Scale of Social Expression – Motor Subscale (MSSE-M), an instrument comprising 64 items, each rated on a five-point Likert scale ranging from 0 (never or very rarely) to 4 (always or very often) [36,37]. The MSSE-M is designed to evaluate eight theoretical dimensions of social competence: (1) initiating and maintaining conversations; (2) refusing requests; (3) receiving compliments; (4) speaking in public; (5) expressing positive emotions; (6) expressing negative emotions; (7) expressing disagreement; and (8) asserting one’s rights. For accurate scoring and to correct for response bias, 38 items were reverse-coded (a score of 4 was recorded as 0, 3 as 1, 1 as 3, and 0 as 4, with 2 remaining unchanged). A factor analysis explored the underlying structure of social skills measured by the MSSE-M, performed to derive meaningful subscale scores. This multivariate statistical technique identified latent dimensions (factors) by examining patterns of correlation among the 64 observed items, thereby grouping them into coherent subscales that reflect specific domains of social competence. The analysis resulted in eight factors, consistent with the instrument’s theoretical structure and corresponding to the eight dimensions listed above. Each factor score, also known as a factorial score, represents an individual’s relative proficiency within that specific domain. We computed factorial scores by summing the scores of items loading onto each respective factor, following the reverse coding where applicable. These scores enable a nuanced interpretation of a participant’s social skills repertoire, both overall (through a global score derived from these factors) and within specific social contexts. Higher factorial scores indicate a broader and more effective repertoire of social skills in the respective domain. For the purpose of this study, scores above the mean of the current study’s sample were considered indicative of a satisfactory level of social competence.

### Statistical analysis

The minimum sample size for this study was calculated using Epitools [38] for detecting a 40 percentage-point difference in the proportions of general social skills repertoire among residents (e.g., assuming a difference between 10% and 50% prevalence). The calculation assumed a 95% confidence level and 80% statistical power. Under these assumptions, a minimum of 25 participants were required for each of the two major residency program categories (medical/multidisciplinary, and veterinary programs), resulting in a total minimum sample size of 50 residents.

To assure internal reliability of the six questionnaires used in the study, the Cronbach’s α coefficient was calculated for all instruments separately, with a cut-off of 0.80 being determined as acceptable internal reliability ([39]; Table 1).

**Fig 2:**
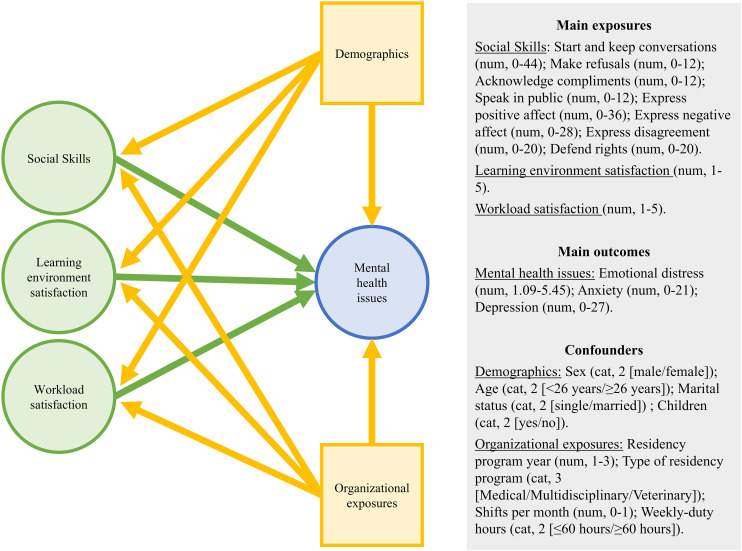
Conceptual causal diagram of hypothesized relationships. This diagram illustrates the assumed causal pathways from demographic and organizational exposure confounders to main exposures (social skills, learning environment satisfaction, and workload satisfaction) and mental health outcomes (emotional distress, anxiety, and depression). Arrows represent hypothesized direct causal effects. Specific variables within each conceptual grouping are detailed.

Multivariable mixed-effects linear regression models were separately built for the three outcomes of interest: emotional distress, anxiety and depression. The social skills total score and the eight factorial scores were included as main explanatory variables of interest. To assess their association and avoid multicollinearity, they were tested in two distinct sets of multivariable models for each outcome, with adjustment for work-related variables (workload and learning environment satisfaction). Additionally, confounders (sex, age, marital status, children, residency program year, type of residency program, shifts per month, and weekly duty) were forced in the models. The conceptual relationship used for visualization of potential confounders are detailed in the causal diagram (Fig 2).

For model building, the first step was assessing linearity between all continuous variables and the outcomes of interest (separately) with linear smooth plots. When the linearity assumption was not met, the variable was dichotomized at the median (if no data was available to inform the categories) or considering the scores categorization as described in Table 1. Second, univariable mixed-effect models (Table 2) were built for each predictor variable. Variables with a *P* value ≤ 0.20 in the univariable analysis were eligible to be tested for inclusion in the final multivariable model. Third, the Spearman correlation method was used to check for collinearity between all independent explanatory variables, with a cutoff of 0.80 to determine highly correlated variables.

**Table 2:** Results from univariable mixed-effect linear regression models for emotional distress, anxiety, and depression in veterinary and multidisciplinary residents. Statistically significant results and trends are bolded.

	Emotional Distress					Anxiety					Depression				
Variable	Coeff.	SE	p	95% CI		Coeff.	SE	p	95% CI		Coeff.	SE	p	95% CI	
Sex (female)	0.32	0.28	0.25	−0.23	0.87	0.12	1.94	0.95	−3.69	3.93	3.89	1.86	**0.04**	0.24	7.54
Age (> 26 years)	0.37	0.23	**0.11**	−0.08	0.83	2.10	1.68	0.21	−1.19	5.39	3.64	1.77	**0.04**	0.16	7.11
Marital Status (married)	0.17	0.28	0.55	−0.38	0.72	3.02	1.90	**0.11**	−0.71	6.76	0.94	2.10	0.65	−3.17	5.05
Children (≥ *1*)	0.09	0.49	0.86	−0.88	1.05	4.12	3.35	0.22	−2.45	10.70	0.86	3.36	0.80	−5.72	7.44
Residency Program year (> 2 year)	0.21	0.23	0.37	−0.25	0.66	0.48	1.60	0.76	−2.65	3.63	0.18	1.63	0.91	−3.01	3.38
Type of Residency Program															
*Veterinary*	−0.26	0.32	0.42	−0.90	0.37	−0.94	2.27	0.68	−5.41	3.51	−2.16	3.14	0.49	−8.31	4.00
*Multidisciplinary*	−0.36	0.31	0.25	−0.97	0.25	−0.73	2.19	0.74	−5.02	3.56	−3.77	3.10	0.22	−9.85	2.31
Shifts per month															
*≤ 5*	−0.02	0.26	0.94	−0.53	0.50	0.41	1.86	0.83	−3.24	4.07	2.68	2.30	0.25	−1.83	7.19
*> 5*	0.32	0.30	0.28	−0.27	0.91	1.94	2.12	0.36	−2.22	6.11	4.57	2.79	**0.10**	−0.90	10.03
Weekly duty (> 60h)	−0.15	0.25	0.55	−0.63	0.33	−0.08	1.70	0.96	−3.42	3.25	0.05	1.93	0.98	−3.74	3.84
Social Skills (≥ 94)	0.00	0.01	0.95	−0.01	0.01	−3.45	1.56	**0.03**	−6.52	−0.37	−3.42	1.61	**0.03**	−6.57	−0.26
*Ability to start and keeping conversations*	−0.01	0.01	0.50	−0.04	0.02	−0.12	0.09	**0.16**	−0.30	0.05	−0.17	0.09	**0.07**	−0.35	0.01
*Ability to make refusals*	0.19	0.23	0.43	−0.27	0.64	0.24	1.61	0.88	−2.92	3.41	1.00	1.61	0.54	−2.15	4.15
*Ability to acknowledge compliments*	0.00	0.39	1.00	−0.77	0.77	−0.39	0.32	0.23	−1.03	0.25	−2.32	2.64	0.38	−7.51	2.86
*Ability to speak in public*	0.14	0.24	0.56	−0.32	0.60	−0.26	0.27	0.34	−0.80	0.27	−0.68	0.27	**0.01**	−1.21	−0.14
*Ability to express positive affect*	−0.08	0.23	0.72	−0.54	0.37	−0.09	0.12	0.47	−0.33	0.15	−0.01	0.13	0.92	−0.27	0.24
*Ability to express negative affect*	−0.28	0.26	0.28	−0.79	0.22	0.19	0.15	0.21	−0.11	0.50	0.21	0.16	**0.18**	−0.10	0.53
*Ability to express disagreement*	−0.01	0.03	0.75	−0.07	0.05	−0.08	0.21	0.67	−0.50	0.32	−0.03	0.21	0.89	−0.45	0.39
*Ability to defend rights*	−0.47	0.29	**0.10**	−1.03	0.09	−2.30	1.99	0.25	−6.20	1.60	−1.35	2.02	0.50	−5.30	2.61
Workload Satisfaction (≥3)	−0.01	0.17	0.95	−0.35	0.32	−2.80	1.60	**0.08**	−5.94	0.33	−1.61	1.68	0.34	−4.89	1.68
Learning Environment Satisfaction (≥3)	−0.46	0.15	**<0.01**	−0.76	−0.16	−4.73	1.46	**<0.01**	−7.60	−1.87	−5.47	0.95	**<0.01**	−7.33	−3.62

Values in bold represent statistical significance at P < 0.2. Age (n = 45) and Weekly duty (n = 48). Categorical variables and their respective references: Sex (male); Age (≤ 26 years); Marital status (single); Children (none); Residency program year (≤ 2); Type of residency program (medical); Shifts per month (none); Weekly-duty hours (≤ 60 hours); Social skills (overall scores ≥ 94); Workload satisfaction (scores ≥ 3); Learning environment satisfaction (scores ≥ 3).

Lastly, we constructed final multivariable mixed-effect linear models. In these models, participants’ institutions were included as a random effect to account for clustering of residents within the same university. We used a manual backwards stepwise approach, starting with all explanatory variables (those with a univariable P ≤ 0.20) and all pre-specified confounding variables (which were forced in the models). Variables were maintained in the final models only if they reached statistical significance (P ≤ 0.05). Additionally, we used the Akaike Information Criterion (AIC) and the Bayesian Information Criterion (BIC) for model selection and comparison, considering the lowest values as a better fit. The normality of residuals was checked using the Best Linear Unbiased Predictors (BLUPs). All analyses were performed using Stata/BE (version 17; StataCorp LLC^®^).

## Results

Fifty-one residents were enrolled in the study, resunting in a response rate of 11.75%. The lowest response rate was for medical residents (3.75%), followed by multidisciplinary (13.51%) and veterinary residents (52.27%). The mean (±SD) age of residents was 28 years ± 4.92 (range 23–48 years, n = 45). Most participants were female (N = 40, 78.43%), single or not cohabiting with others (N = 40, 78.43%), and with no children (N = 48, 94.12%). Table 3 presents the detailed descriptive analysis of the study participants, including sex, age (n = 45), marital status, children, residency program year, type of residency program, shifts per month, and weekly duty (n = 48). The general prevalence of emotional distress, anxiety, and depression in this study, for the three residency types, was 86.27% (n = 44), 58.82% (n = 30), and 56.86% (n = 29), respectively.

**Table 3:** Descriptive analysis of veterinary, medical, and multidisciplinary residents.

Variable	N	Frequency
Sex		
*Male*	11	21.57%
*Female*	40	78.43%
Age		
*≤ 26 years*	22	48.89%
*> 26 years*	23	51.11%
Marital Status		
*Single/does not cohabit with others*	40	78.43%
*Married/cohabiting with others*	11	21.57%
Children		
*none*	48	94.12%
≥ *1*	3	5.88%
Residency Program year		
*1st year*	27	52.94%
*2nd or 3rd year*	24	47.06%
Type of Residency Program		
*Medical*	10	19.61%
*Multidisciplinary*	18	35.29%
*Veterinary*	23	45.10%
Shifts per month		
*none*	20	39.22%
*≤ 5*	19	37.25%
*> 5*	12	23.53%
Weekly duty		
*≤ 60 hours*	29	60.42%
*> 60 hours*	19	39.58%

Age (n = 45) and Weekly duty (n = 48). For ‘Shifts per month’ = none, a shift was not required by the program specialty.

**Fig 3:**
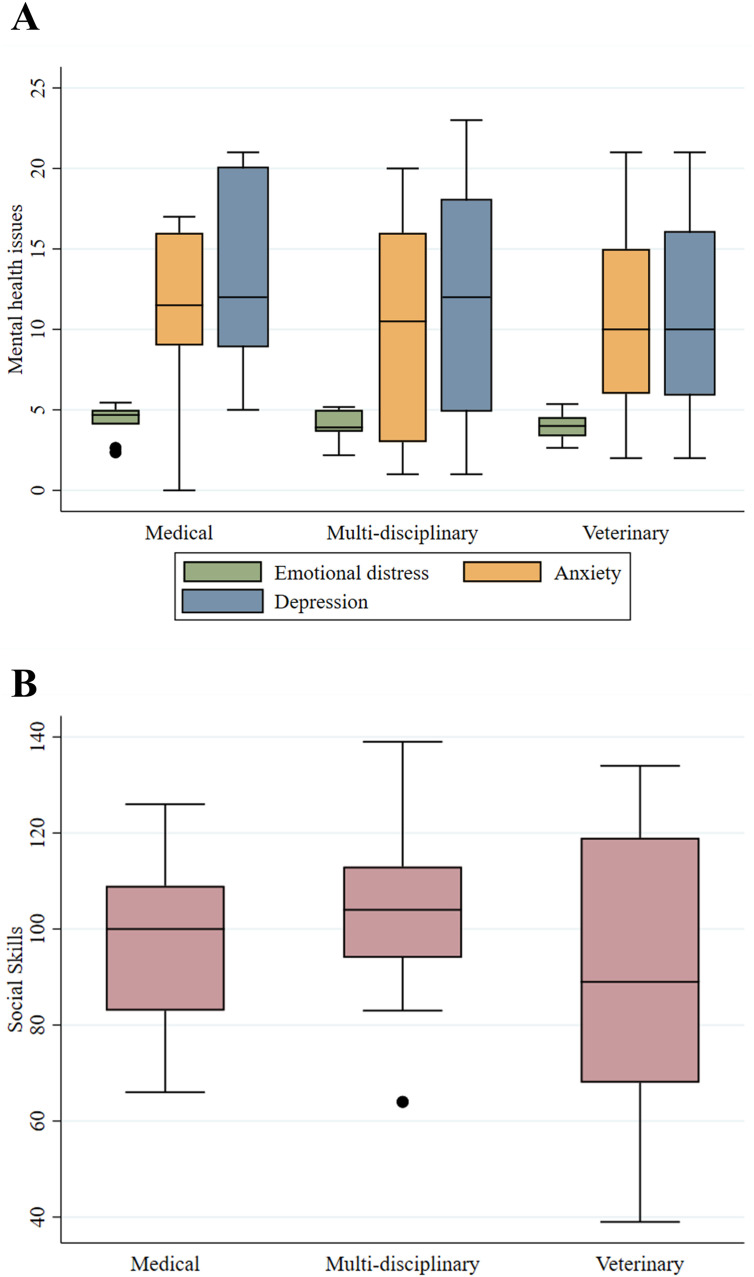
A) Scores of mental health issues by type of residency program and B) Total scores of social skills by type of residency program.

Medical residents presented higher medians of emotional distress, anxiety, and depression when compared with the multidisciplinary and veterinary residents (Fig 3A). However, the multivariate analysis found no significant difference between the mental health conditions examined and type of residency program. Briefly, for the scales presented in Figure 3: higher scores on GAD-7 and PHQ-9 indicate greater anxiety and depression symptoms, respectively (with scores above 10 on both suggesting clinically relevant levels). Conversely, lower scores on the emotional distress scale denote less stress. For social skills (MSSE-M), higher scores reflect a broader repertoire, and scores above the study sample’s mean indicate satisfactory competence (Fig 3B). Higher scores on the workload and learning environment satisfaction scales indicate greater satisfaction.

Veterinary residents showed higher scores on “learning environment satisfaction” (65.21%, n = 15), but in contrast, only 21.73% (n = 5) for workload satisfaction. For comparison, medical residents reported 40% (n = 4) for learning environment satisfaction and 40% (n = 4) for workload satisfaction. Multidisciplinary residents reported 44.44% (n = 8) for learning environment satisfaction and 61.11% (n = 11) for workload satisfaction.

**Fig 4:**
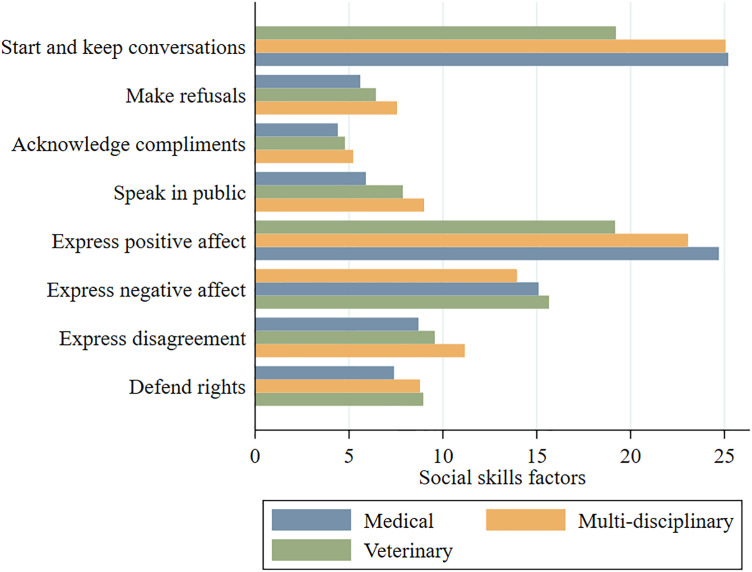
Total scores of social skills subscales by the type of residency program.

Overall, 60.78% (n = 31) of residents presented a “satisfactory” (above average of scores) repertoire of social skills in the total scores. Within the type of residency program, the multidisciplinary residents had the higher total scores of social skills (Fig 3B) and also presented the higher means of workload satisfaction (n = 18, mean 3.17, SD = .40). Among the factorial social skills subscales, “Start and keeping conversations” and “Express positive affect” showed the highest scores across residents of all three programs (Fig 4).

The final multivariable model (Table 4) for emotional distress retained two significant associated factors: the “ability to defend rights” social skills subscale (score ≥ 13, which showed a protective effect, reducing distress; Coeff. −0.58, 95% CI [−1.08, −0.08], P = .02); and “learning environment satisfaction” (score ≥ 3, where higher satisfaction associated with reduced distress; Coeff. −0.48, 95% CI [−0.77, −0.19], P = < .01). For anxiety and depression, both final models retained the total score for the “social skills” variable (score ≥ 94, where higher social skills scores associated with reduced anxiety and depression; Coeff. −3.14, 95% CI [−6.31, 0.03], P = .05 and Coeff. −3.92, 95% CI [−6.28, −1.57], P = ≤.01, respectively), along with “learning environment satisfaction” (score ≥ 3, where higher satisfaction was associated with reduced anxiety and depression; Coeff. −5.96, 95% CI [−9.20, −2.72], P ≤ .01 for anxiety and Coeff. −5.40, 95% CI [−6.99, −3.82], P ≤ .01 for depression). Additionally, being married (vs. single/does not cohabit) increased anxiety scores by approximately 5 units (Coeff. 4.97, 95% CI [0.47, 9.46], P = .03). Lastly, several demographic variables were significant associated factors for depression in the final multivariable model: identifying as a female (vs. male, associated with increased depression scores; Coeff. 5.49, 95% CI [2.64, 8.34], P = ≤.01), having more than 26 years of age (vs. ≤ 26 years, associated with increased depression scores; Coeff. 2.78, 95% CI [0.29, 5.28], P = .03), having more than one child (vs. none, associated with decreased depression scores; Coeff. −5.72, 95% CI [−10.41, −1.04], P = .02), and doing between one and five shifts per month (vs. none, associated with increased depression scores; Coeff. 3.21, 95% CI [0.39, 6.03], P = .03).

**Table 4:** Results from final multivariable mixed-effect linear regression model for emotional distress, anxiety and depression in veterinary and multidisciplinary residents.

	Emotional Distress					Anxiety					Depression				
Variable	Coeff.	Se	p	95% CI		Coeff.	Se	p	95% CI		Coeff.	Se	p	95% CI	
Sex (female)	0.32	0.27	0.23	−0.21	0.85	−1.07	1.96	0.58	−4.91	2.76	5.49	1.45	**<0.01**	2.64	8.34
Age (> 26 years)	0.19	0.24	0.43	−0.29	0.67	−0.48	1.72	0.78	−3.84	2.89	2.78	1.27	**0.03**	0.29	5.28
Marital Status (married)	0.21	0.32	0.51	−0.41	0.83	4.97	2.29	**0.03**	0.47	9.46	3.25	1.72	0.06	−0.12	6.61
Children (≥ *1*)	−0.62	0.45	0.17	−1.51	0.27	−0.64	3.18	0.84	−6.87	5.59	−5.72	2.39	**0.02**	−10.41	−1.04
Residency Program year (>2 years)	0.14	0.22	0.52	−0.29	0.58	0.19	1.62	0.91	−2.98	3.37	−1.07	1.18	0.37	−3.39	1.25
Type of Residency Program															
*Veterinary*	−0.14	0.41	0.73	−0.94	0.66	−0.86	2.92	0.77	−6.58	4.86	0.60	2.19	0.78	−3.69	4.90
*Multidisciplinary*	0.02	0.45	0.97	−0.86	0.89	−1.40	3.37	0.68	−8.00	5.20	0.30	2.51	0.91	−4.62	5.22
Weekly duty (> 60h)	0.06	0.28	0.82	−0.49	0.62	0.35	2.06	0.87	−3.69	4.39	0.01	1.50	1.00	−2.94	2.95
Shifts per month															
*≤ 5*	0.35	0.27	0.19	−0.18	0.87	2.38	1.96	0.22	−1.46	6.21	3.21	1.44	**0.03**	0.39	6.03
*> 5*	0.50	0.44	0.26	−0.37	1.36	1.76	3.24	0.59	−4.59	8.12	4.09	2.38	0.09	−0.57	8.74
Social Skills (≥ 94)						−3.14	1.62	**0.05**	−6.31	0.03	−3.92	1.20	**<0.01**	−6.28	−1.57
*Ability to defend rights (≥13)*	−0.58	0.26	**0.02**	−1.08	−0.08										
Learning Environment Satisfaction (≥ 3)	−0.48	0.15	**<0.01**	−0.77	−0.19	−5.96	1.65	**<0.01**	−9.20	−2.72	−5.40	0.81	**<0.01**	−6.99	−3.82

Values in bold represent statistical significance at P < .05. Age (n = 45) and Weekly duty (n = 48). Categorical variables and their respective references: Sex (male); Age (≤ 26 years); Marital status (single); Children (none); Residency program year (≤ 2); Type of residency program (medical); Shifts per month (none); Weekly-duty hours (≤ 60 hours); Social skills (overall scores ≥ 94); Social skills – ability to defend rights (scores ≥ 13); Learning environment satisfaction (scores ≥ 3).

## Discussion

We observed a high prevalence of emotional distress (86.27%), anxiety (58.82%), and depression (56.86%) in our results, in line with other studies that analyzed residents [28,40–42]. It is important to contextualize these findings, as the data were collected between August and November 2019, prior to the COVID-19 pandemic. Consequently, these results reflect the pre-pandemic mental health situation of residents and may not be directly comparable to the current, post-COVID context, where professional demands and mental health dynamics have been profoundly transformed [43].

Surprisingly, the type of residency program didn’t show significant differences regarding the mental health issues in the models, but medical residents descriptively presented higher median scores for emotional distress, anxiety, and depression when compared to multidisciplinary and veterinary residents (Fig 3A). The high prevalence of emotional distress and anxiety observed in our sample is in line with international studies, underscoring the systemic and global nature of these stressors [9].

Physicians and veterinarians are professionals that are vulnerably exposed to high levels of stress, anxiety and depression, faced by work stressors such as high levels of responsibility, exposure to death and grieving, and high workload [24,41]. Research has recently explored the mental health of veterinarians since they have higher rates and risk of suicide deaths when compared to the general population and physicians [44–47]. Moral dilemmas, such as legal or financial constraints, veterinarians can specifically face more commonly, forcing them to make difficult clinical decisions such as performing euthanasia, even if other treatment options are available.

The recurring feeling of regret, and avoidance to acknowledge feelings and distress can lead these health professionals to seek relief in drug substances and alcohol [48,49]. In contrast with many studies that have shown the high prevalence of anxiety and depression in veterinary residents, our study’s descriptive findings indicated that medical residents had higher median scores for anxiety and depression when compared to residents from the veterinary residency program. This could be explained by the differences between the three programs regarding the institutional policies, number of shifts per month, workload, and responsibilities.

Descriptive analysis showed that multidisciplinary residents had numerically higher scores for social skills and workload satisfaction compared to medical and veterinary residents. While these differences were not statistically significant in our models, a plausible explanation for the observed trend could be related to the mandatory regulations of this type of residency program, restricting the residents to strictly follow a specific number of shifts per month, and have a workload of 60 hours maximum per week [50]. Other programs, such as some medical residencies in our study, reported varying regulations and higher workloads, with some residents working more than 100 hours per week and doing more than 8 shifts per month.

In the final multivariable models, the significant associated factors with depression were being female, having more than 26 years of age, having more than one child, and doing between one and five shifts per month. Being married increased the anxiety scores significantly when compared to being single, although it has been suggested that being married could have a protective psychological effect [51]. The role of workload is also crucial in resident well-being; for instance, De Mélo Silva Júnior et al. (2022)[40] found that duty-hours were associated with lack of work-life balance and increased anxiety and headache in medical residents. While our multivariable models did not find a significant association between shifts per month and anxiety, our findings for depression highlight the impact of workload as a stressor.

The multivariable models identified the variable “learning environment satisfaction” as an important associated factor for emotional distress, anxiety, and depression. Numerous studies have suggested that a positive and supportive learning environment contributes to residents’ well-being, job satisfaction, performance, quality of resident education, and overall resident’s mental health [2,17,52,53]. Although our models did not find a significant association for workload satisfaction, some studies have highlighted the importance of manageable workload; for example, one study examining physician residents found that increased work hours were associated with depressive symptoms [5]. This indicates that while workload is generally recognized as a factor [40,54], in our study, the perceived satisfaction with the learning environment emerged as a more critical associated factor for mental health outcomes.

Finally, our study found that a good repertoire of social skills played a protective role for emotional distress, anxiety, and depression. Specifically, for emotional distress, the “ability to defend rights” social skills subscale emerged as a significant associated protective factor (Table 4). This finding is particularly noteworthy as it suggests that the capacity for self-advocacy and setting boundaries plays a crucial role in mitigating emotional distress among residents. The ‘ability to defend rights’ encompasses assertiveness, the capacity to express one’s needs, and to stand up for oneself in challenging environments common in residency training, such as high workload or demanding interpersonal interactions [55]. This skill may empower residents to manage stress, avoid burnout, and maintain psychological well-being by allowing them to navigate professional demands more effectively and prevent situations that could otherwise lead to distress [56]. For depression, higher overall social skills scores were significantly protective (P ≤ .01). For anxiety, this protective association was also observed, reaching the threshold of statistical significance (P = .05), aligning with other studies [24] that suggest a broader repertoire of social competencies reduces emotional exhaustion and promotes mental health. These abilities are closely linked to effective communication, assertiveness, and positive emotions, thereby contributing to the creation of a supportive and psychologically healthy environment for residents [57].

Studies have shown the expression of positive affect to be closely related to experiencing pleasurable emotions, such as feelings of satisfaction, achievement, motivation, and desire for social connection [58]. In line with our findings, Dominguez-Espinosa et al. (2022)[58] emphasized the importance of cultivating a culture of gratitude and appreciation among colleagues to address the reported lack of positive affect. Therefore, incorporating social skills training into educational programs can be beneficial for resident doctors, as suggested by Yutani et al., (2016)[59], and could possibly be extrapolated to the other disciplines. By enhancing their social skills to navigate challenging situations, residents can improve their ability to provide high-quality medical care, reduce stress levels, and safeguard their mental health [59,60].

### Strengths and limitations

This study offers several notable strengths. To the best of our knowledge, it is the first to assess the association between social skills, emotional distress, anxiety, and depression specifically within Brazilian medical, multidisciplinary, and veterinary residents. Our findings provide valuable insights into the mental health of residents, examining their relationship with social skills and various work-related factors. A unique contribution of this study is the comparative analysis across these three distinct categories of health professionals, which allows for a nuanced understanding of their differing mental health profiles. By investigating these diverse residency programs, we identified potential risk factors for stress, anxiety, and depression, thereby offering a comprehensive perspective on these prevalent issues. Furthermore, our research highlights the crucial role of social skills and satisfaction with the learning environment in promoting the mental well-being of medical, multidisciplinary, and veterinary residents, enriching broader discussions on resident wellness. Finally, the application of multivariable analysis, carefully accounting for demographic variables as potential confounders, enhanced the reliability and accuracy of our findings. This robust modeling approach strengthened the validity of the study by mitigating bias and providing a clearer understanding of the relationships between the exposure and outcome variables of interest.

Despite these strengths, it is essential to acknowledge the study’s limitations. Firstly, the relatively modest sample size and the inclusion of only a small proportion of Brazilian medical, multidisciplinary, and veterinary residents necessitates careful consideration when generalizing the findings to broader resident populations. Crucially, the reliance on a convenience sampling strategy coupled with a notably low response rate (11.75%) poses significant limitations to the external validity and generalizability of the findings. This methodological approach introduces an inherent risk of selection bias, as participants were not randomly selected and those who chose to respond may differ systematically from non-respondents. Consequently, the representativeness of the sample relative to the broader population of health residency professionals in Brazil is limited, particularly due to the lack of national geographical representation, as participants were drawn from only five institutions. Likewise, the majority of our respondents were females, which may have impacted study findings. These factors should be carefully weighed during the interpretation of the results. It is also important to acknowledge that only five institutions were used as random effects, which may produce unstable model estimates.

Secondly, recruiting participants exclusively online may have further discouraged response rates. Residents might have been hesitant to participate due to the questionnaire’s length and the sensitive nature of the topic, as previous studies indicate that online surveys are generally associated with lower participation rates compared to mail or in-person questionnaires [61–63]. While efforts were made to address potential response bias, alternative data collection methods were not feasible given the geographical distribution of the five participating institutions.

Thirdly, a limitation of this study is the absence of data on participants’ socioeconomic conditions or their access to formal psychosocial support mechanisms. Such variables could serve as important confounders or mediators of mental health outcomes and their omission may limit a comprehensive understanding of the observed associations. Lastly, the use of a cross-sectional design limits our ability to establish causal relationships among the variables, only allowing for the identification of associations. It is also important to note that using a causal diagram and univariable analysis as a screening approach represents only one possible strategy for analyzing the data. Future work could explore and compare alternative exploratory techniques to assess variables to investigate whether they yield additional insights.

## Conclusions

This study highlights the association between social skills and mental health concerns among Brazilian medical, multidisciplinary, and veterinary residents, while considering demographic and work-related satisfaction variables. Our findings reveal a high prevalence of mental health issues among residents in these programs, with learning environment satisfaction emerging as a significant associated factor in the final multivariable analysis for all three programs. Notably, a strong repertoire of social skills, particularly in overall scores, exhibited a protective association against mental health issues, confirming our initial hypothesis. These results emphasize the importance of incorporating social skills training within residency programs, specifically focusing on assertiveness, as exemplified by the ability to defend rights and express positive affect, to safeguard residents’ mental health. Furthermore, we recommend fostering a supportive learning environment and promoting the training of social skills, especially the abilities of communication and assertiveness, during residency programs as a routine tool for mental health promotion and safe work environment for residents in training. Based on these insights, it is also important to consider the development of institutional policies aimed at promoting social skills and overall well-being in residency settings. For future research, longitudinal studies would be valuable to further explore these relationships and establish causal effects.

## Supporting information

S1 File Complete study questionnaire and validated instrument scales. (DOCX)

## References

[1] Rosa SD, Lopes RE. Residência multiprofissional em saúde e pós-graduação lato sensu no Brasil: apontamentos históricos. Trab educ saúde. 2009;7(3):479–98. doi: 10.1590/s1981-77462009000300006

[2] Weiss KB, Bagian JP, Nasca TJ. The clinical learning environment: the foundation of graduate medical education. JAMA. 2013;309(16):1687–8. doi: 10.1001/jama.2013.1931 23613072

[3] Furr MO, Raczkoski BM. A decision-tree model of career choice for veterinarians in clinical residency programs. J Am Vet Med Assoc. 2022;260(5):549–58. doi: 10.2460/javma.21.02.0075 34986122

[4] Gupta S, Higgins S, Torre D. Wellbeing and burnout in residency. J Gen Intern Med. 2022;37(9):2137–8. doi: 10.1007/s11606-022-07663-6 35606642 PMC9126691

[5] Pereira-Lima K, Gupta RR, Guille C, Sen S. Residency program factors associated with depressive symptoms in internal medicine interns: A prospective cohort study. Acad Med. 2019;94(6):869–75. doi: 10.1097/ACM.0000000000002567 30570500 PMC6538448

[6] Fried EI, Nesse RM, Guille C, Sen S. The differential influence of life stress on individual symptoms of depression. Acta Psychiatr Scand. 2015;131(6):465–71. doi: 10.1111/acps.12395 25650176 PMC4428974

[7] Jaworski JL, Thompson LA, Weng H-Y. Quality of life of veterinary residents in AVMA-Recognized Veterinary Specialty Organizations using the WHOQOL-BREF instrument. PLoS One. 2022;17(5):e0268343. doi: 10.1371/journal.pone.0268343 35551334 PMC9098004

[8] Sen S, Kranzler HR, Krystal JH, Speller H, Chan G, Gelernter J, et al. A prospective cohort study investigating factors associated with depression during medical internship. Arch Gen Psychiatry. 2010;67(6):557–65. doi: 10.1001/archgenpsychiatry.2010.41 20368500 PMC4036806

[9] Obeng Nkrumah S, Adu MK, Agyapong B, da Luz Dias R, Agyapong VIO. Prevalence and correlates of depression, anxiety, and burnout among physicians and postgraduate medical trainees: a scoping review of recent literature. Front Public Health. 2025;13:1537108. doi: 10.3389/fpubh.2025.1537108 40697832 PMC12279716

[10] McPhetridge JB, Scharf VF, Dickson R, Thieman KM, Oblak ML, Regier PJ, et al. Veterinary house officer perceptions of dimensions of well-being during postgraduate training. J Am Vet Med Assoc. 2022;260(11):1369–76. doi: 10.2460/javma.21.05.0233 35429376

[11] Monticelli P, Seymour C, Adami C. Risk of burnout and depression: A survey of veterinary anaesthesia specialists in-training during COVID-19. Vet Anaesth Analg. 2023;50(4):325–32. doi: 10.1016/j.vaa.2023.04.001 37179142 PMC10102702

[12] Guille C, Clark S, Amstadter AB, Sen S. Trajectories of depressive symptoms in response to prolonged stress in medical interns. Acta Psychiatr Scand. 2014;129(2):109–15. doi: 10.1111/acps.12137 23581856 PMC4073633

[13] Grant F, Guille C, Sen S. Well-being and the risk of depression under stress. PLoS One. 2013;8(7):e67395. doi: 10.1371/journal.pone.0067395 23840872 PMC3698120

[14] de Oliveira GS, Chang R, Fitzgerald PC, Almeida MD, Castro-Alves LS, Ahmad S, et al. The prevalence of burnout and depression and their association with adherence to safety and practice standards: A survey of United States anesthesiology trainees. Anesth Analg. 2013;117(1):182–93. doi: 10.1213/ANE.0b013e3182917da9 23687232

[15] Fahrenkopf AM, Sectish TC, Barger LK, Sharek PJ, Lewin D, Chiang VW, et al. Rates of medication errors among depressed and burnt out residents: Prospective cohort study. BMJ. 2008;336(7642):488–91. doi: 10.1136/bmj.39469.763218.BE 18258931 PMC2258399

[16] West CP, Tan AD, Habermann TM, Sloan JA, Shanafelt TD. Association of resident fatigue and distress with perceived medical errors. JAMA. 2009;302(12):1294–300. doi: 10.1001/jama.2009.1389 19773564

[17] Jennings ML, Slavin SJ. Resident wellness matters: Optimizing resident education and wellness through the learning environment. Acad Med. 2015;90(9):1246–50. doi: 10.1097/ACM.0000000000000842 26177527

[18] Nasca TJ, Weiss KB, Bagian JP. Improving clinical learning environments for tomorrow’s physicians. N Engl J Med. 2014;370(11):991–3. doi: 10.1056/NEJMp1314628 24467307

[19] Li J, Weigl M, Glaser J, Petru R, Siegrist J, Angerer P. Changes in psychosocial work environment and depressive symptoms: A prospective study in junior physicians. Am J Ind Med. 2013;56(12):1414–22. doi: 10.1002/ajim.22246 24038041

[20] Sakata Y, Wada K, Tsutsumi A, Ishikawa H, Aratake Y, Watanabe M, et al. Effort-reward imbalance and depression in Japanese medical residents. J Occup Health. 2008;50(6):498–504. doi: 10.1539/joh.l8043 18946190

[21] Weigl M, Hornung S, Petru R, Glaser J, Angerer P. Depressive symptoms in junior doctors: A follow-up study on work-related determinants. Int Arch Occup Environ Health. 2012;85(5):559–70. doi: 10.1007/s00420-011-0706-8 21956449

[22] Wilson AN, Dow E, Hanami D, Vasa M, Billimek J. Encouraging mental health care in family medicine residents. PRiMER. 2022;6:24. doi: 10.22454/PRiMER.2022.147530 36119905 PMC9477719

[23] Duijn C, Bok H, ten Cate O, Kremer W. Qualified but not yet fully competent: Perceptions of recent veterinary graduates on their day‐one skills. Veterinary Record. 2020;186(7):216–216. doi: 10.1136/vr.105329 31767696

[24] Pereira-Lima K, Loureiro SR. Burnout, anxiety, depression, and social skills in medical residents. Psychol Health Med. 2015;20(3):353–62. doi: 10.1080/13548506.2014.936889 25030412

[25] Treister-Goltzman Y, Samson T, Rosenberg R, Granek-Catarivas M, Gaver A, Alperin M, et al. Burnout among family medicine residents: A cross-sectional nationwide study. Isr J Health Policy Res. 2024;13(1):5. doi: 10.1186/s13584-024-00591-2 38279151 PMC10811917

[26] Barfield AD, Adamantos S. Looking after interns and residents. Veterinary Record. 2019;184:99–100. 10.1136/vr.l1201 30655405

[27] Rodrigues H, Cobucci R, Oliveira A, Cabral JV, Medeiros L, Gurgel K, et al. Burnout syndrome among medical residents: A systematic review and meta-analysis. PLoS One. 2018;13(11):e0206840. doi: 10.1371/journal.pone.0206840 30418984 PMC6231624

[28] Rotta DS, Pinto MH, Lourenção LG, Teixeira PR, Gonsalez EG, Gazetta CE. Anxiety and depression levels among multidisciplinary health residents. Rev Rene. 2016;17(3):372. doi: 10.15253/2175-6783.2016000300010

[29] Cheade M de FM, Frota OP, Loureiro MDR, Quintanilha ACF. Residência multiprofissional em saúde: A busca pela integralidade. Cogitare Enferm. 2013;18(3). doi: 10.5380/ce.v18i3.46360

[30] Guido L de A, Goulart CT, da Silva RM, Lopes LFD, Ferreira EM. Stress and burnout among multidisciplinary residents. Rev Lat Am Enfermagem. 2012;20(6):1064–71. doi: 10.1590/s0104-11692012000600008 23258719

[31] Pereira-Lima K, Silva-Rodrigues APC, Marucci FAF, Osório F de L, Crippa JA, Loureiro SR. Cross-cultural adaptation and psychometric assessment of a Brazilian-Portuguese version of the Resident Questionnaire. PLoS One. 2018;13(9):e0203531. doi: 10.1371/journal.pone.0203531 30180216 PMC6122823

[32] Seelig CB, DuPre CT, Adelman HM. Development and validation of a scaled questionnaire for evaluation of residency programs. South Med J. 1995;88(7):745–50. doi: 10.1097/00007611-199507000-00010 7597480

[33] Spitzer RL, Kroenke K, Williams JBW, Löwe B. A brief measure for assessing generalized anxiety disorder: the GAD-7. Arch Intern Med. 2006;166(10):1092–7. doi: 10.1001/archinte.166.10.1092 16717171

[34] Santos IS, Tavares BF, Munhoz TN, Almeida LSP, Silva NTB, Tams BD, et al. Sensibilidade e especificidade do Patient Health Questionnaire-9 (PHQ-9) entre adultos da população geral. Cad Saúde Pública. 2013;29(8):1533–43. doi: 10.1590/s0102-311x2013001200006 24005919

[35] Kroenke K, Spitzer RL, Williams JB. The PHQ-9: Validity of a brief depression severity measure. J Gen Intern Med. 2001;16(9):606–13. doi: 10.1046/j.1525-1497.2001.016009606.x 11556941 PMC1495268

[36] Caballo VE. La multidimensionalidad conductual de habilidades sociales: Propiedades psicométricas de una medida de autoinforme, la EMES-M. Psicologia Conductual. 1993;1:221–31.

[37] Pereira AS, Dutra-Thomé L, Koller SH. Propriedades psicométricas da escala multidimensional de expressão social -parte motora (EMES-M) em uma amostra brasileira. Aval Psicol. 2017;17(01). doi: 10.15689/ap.2017.1701.14.13651

[38] Sergeant ESG. Epitools Epidemiological Calculators. Australia: Ausvet; 2018.

[39] Gliem JA, Gliem RR. Calculating, interpreting, and reporting Cronbach’s alpha reliability coefficient for Likert type scales. Columbus, Ohio: The Ohio State University. 2003. https://hdl.handle.net/1805/344

[40] de Mélo Silva Júnior ML, Valença MM, Rocha-Filho PAS. Individual and residency program factors related to depression, anxiety and burnout in physician residents - a Brazilian survey. BMC Psychiatry. 2022;22(1):272. doi: 10.1186/s12888-022-03916-0 35436910 PMC9016975

[41] Golob A, Beste LA, Stern M, Johnson K. Emotional distress among physician residents and fellows: An observational study of trainees seeking counseling visits. Acad Psychiatry. 2018;42(1):25–30. doi: 10.1007/s40596-017-0740-2 28608232

[42] Raimo J, LaVine S, Spielmann K, Akerman M, Friedman KA, Katona K, et al. The correlation of stress in residency with future stress and burnout: A 10-year prospective cohort study. J Grad Med Educ. 2018;10(5):524–31. doi: 10.4300/JGME-D-18-00273.1 30386477 PMC6194879

[43] Caminiti M, Mercogliano M, Cussotto F, Briganti GL, Genovese D, Priano W, et al. Study protocol for the residents’ mental health investigation, a dynamic longitudinal study in Italy (ReMInDIt). Healthcare (Basel). 2024;12(10):1020. doi: 10.3390/healthcare12101020 38786430 PMC11121525

[44] Glaesmer H, Bahramsoltani M, Schwerdtfeger K, Spangenberg L. Euthanasia distress and fearlessness about death in german veterinarians. Crisis. 2021;42(1):71–7. doi: 10.1027/0227-5910/a000689 32431195

[45] Milner AJ, Niven H, Page K, LaMontagne AD. Suicide in veterinarians and veterinary nurses in Australia: 2001-2012. Aust Vet J. 2015;93(9):308–10. doi: 10.1111/avj.12358 26313208

[46] Kelly JE. Notes From the Field. The Historian. 2019;81(2):209–12. doi: 10.1111/hisn.13131

[47] Tomasi SE, Fechter-Leggett ED, Edwards NT, Reddish AD, Crosby AE, Nett RJ. Suicide among veterinarians in the United States from 1979 through 2015. J Am Vet Med Assoc. 2019;254(1):104–12. doi: 10.2460/javma.254.1.104 30668293 PMC6417412

[48] Blum NR. Professional development groups help physicians; why not veterinarians?. J Am Vet Med Assoc. 2018;253(6):704–8. doi: 10.2460/javma.253.6.704 30179098

[49] Dalum HS, Tyssen R, Hem E. Prevalence and individual and work-related factors associated with suicidal thoughts and behaviours among veterinarians in Norway: A cross-sectional, nationwide survey-based study (the NORVET study). BMJ Open. 2022;12(1):e055827. doi: 10.1136/bmjopen-2021-055827 34980627 PMC8724721

[50] Secretaria de Educação Superior. Resolução Comissão Nacional de Residência Multiprofissional em Saúde. 2012. http://portal.mec.gov.br/index.php?option=com_docman&view=download&alias=15448-resol-cnrms-n2-13abril-2012&Itemid=30192

[51] Perret JL, Best CO, Coe JB, Greer AL, Khosa DK, Jones-Bitton A. Association of demographic, career, and lifestyle factors with resilience and association of resilience with mental health outcomes in veterinarians in Canada. J Am Vet Med Assoc. 2020;257(10):1057–68. doi: 10.2460/javma.2020.257.10.1057 33135980

[52] Pelzer JM, Hodgson JL, Werre SR. Veterinary students’ perceptions of their learning environment as measured by the Dundee Ready Education Environment Measure. BMC Res Notes. 2014;7:170. doi: 10.1186/1756-0500-7-170 24661621 PMC3987886

[53] Quansah F, Hagan JE, Sambah F, Frimpong JB, Ankomah F, Srem-Sai M, et al. Perceived safety of learning environment and associated anxiety factors during COVID-19 in Ghana: Evidence from physical education practical-oriented program. Eur J Investig Health Psychol Educ. 2022;12(1):28–41. doi: 10.3390/ejihpe12010003 35049532 PMC8774500

[54] Pohl R, Botscharow J, Böckelmann I, Thielmann B. Stress and strain among veterinarians: A scoping review. Ir Vet J. 2022;75(1):15. doi: 10.1186/s13620-022-00220-x 35729648 PMC9209636

[55] Gutgeld-Dror M, Laor N, Karnieli-Miller O. Assertiveness in physicians’ interpersonal professional encounters: A scoping review. Med Educ. 2024;58(4):392–404. doi: 10.1111/medu.15222 37725417

[56] Ghannam J, Afana A, Ho EY, Al-Khal A, Bylund CL. The impact of a stress management intervention on medical residents’ stress and burnout. International Journal of Stress Management. 2020;27(1):65–73. doi: 10.1037/str0000125

[57] R K, Sujathamalini J, Gunasekaran K, Pandimeenal A. Correlation study on assertiveness and empathy among young adults. Int J Psychol Res. 2025;7(1):45–8. doi: 10.33545/26648903.2025.v7.i1a.77

[58] Dominguez-Espinosa ADC, Montes de Oca-Mayagoitia SI, Sáez-Jiménez AP, de la Fuente-Zepeda J, Monroy Ramírez de Arellano L. The moderating role of sociodemographic and work-related variables in burnout and mental health levels of Mexican medical residents. PLoS One. 2022;17(9):e0274322. doi: 10.1371/journal.pone.0274322 36112642 PMC9481024

[59] Yutani M, Takahashi M, Takizawa T, Miyaoka H. Social skills training improves clinical residents’ communication skills. Kitasato Medical Journal. 2016;1:53–9.

[60] Mueser KT, Bellack AS. Social skills training: Alive and well?. Journal of Mental Health. 2007;16(5):549–52. doi: 10.1080/09638230701494951

[61] Suppan M, Suppan L, Beckmann TS, Samer CF, Savoldelli GL. Enhancing response rates in web-based surveys: The impact of direct participant contact. Healthcare (Basel). 2024;12(14):1439. doi: 10.3390/healthcare12141439 39057582 PMC11276369

[62] Daikeler J, Bošnjak M, Lozar Manfreda K. Web versus other survey modes: An Updated and extended meta-analysis comparing response rates. Journal of Survey Statistics and Methodology. 2019;8(3):513–39. doi: 10.1093/jssam/smz008

[63] Zhang X, Kuchinke L, Woud ML, Velten J, Margraf J. Survey method matters: Online/offline questionnaires and face-to-face or telephone interviews differ. Computers in Human Behavior. 2017;71:172–80. doi: 10.1016/j.chb.2017.02.006

