# Supplementary material for: Association between social skills and mental health outcomes in Health Science Residents in Brazil
Source: PLoS One. 2026 Feb 18;21(2):e0341773. doi: 10.1371/journal.pone.0341773 (PMC12915928; doi:10.1371/journal.pone.0341773)
Supplement: S1 File — (DOCX) [file pone.0341773.s001.docx]

**Supporting Information S1**

**S1 Text. Complete study questionnaire and validated instrument scales.**

**SOCIODEMOGRAPHIC AND PROFESSIONAL ACTIVITIES QUESTIONNAIRE**

Q01) Sex (male/female):

Q02) Age

Q03) Marital status (single/married):

Q04) Do you have children? (yes/no)

Q05) If yes, how many children do you have?

Q06) Who do you live with?

Q07) Which university did you graduate from?

Q08) Did you complete an extracurricular internship during your graduation? (yes/no)

Q09) Current university:

Q10) Current residency program (Medical/ Multidisciplinary/ Veterinary):

Q11) Current residency program area:

Q12) Year of residency:

Q13) Direct access program (yes/no):

Q14) Duration of previous program:

Q15) Does the program require regular shifts? (yes/no):

Q16) If yes, what is the number of regular residency shifts per month?

Q17) Do you perform extra shifts outside the residency? (yes/no)

Q18) Number of extra shifts outside the residency per month:

Q19) Weekly workload dedicated solely to residency activities:

Q20) Weekly workload including all professional activities:

**RESIDENT QUESTIONNAIRE (RQ)**

Seelig CB, Dupre CT, Adelman HM. Development and Validation of a Scaled Questionnaire for Evaluation of Residency Programs: Southern Medical Journal. 1995 Jul;88(7):745–50.

Please complete the following questionnaire which aims to identify information that can help improve medical and multi-disciplinary residency programs. Answer all the questions below, using the scale provided, selecting the answer that best reflects your assessment.

1 - Strongly disagree

2 - Disagree

3 - Neither disagrees nor agrees

4 - Agree

5 - Strongly agree

| **Questions** | **1** | **2** | **3** | **4** | **5** |
| --- | --- | --- | --- | --- | --- |
| I receive appropriate feedback from the supervisors at the appropriate time. |  |  |  |  |  |
| The deadline requirements for the tasks are reasonable and allow me to do my job. |  |  |  |  |  |
| I often feel frustrated. |  |  |  |  |  |
| Hospital support services (examples: logistics, cleaning, auxiliaries and technicians) are sufficient to help me take care of my patients. |  |  |  |  |  |
| I usually enjoy life. |  |  |  |  |  |
| Scheduled clinical meetings are often valuable learning experiences. |  |  |  |  |  |
| I often feel irritated by things that happen at work. |  |  |  |  |  |
| The shift scale is very heavy. |  |  |  |  |  |
| Internship rotations in wards are often a good learning experience. |  |  |  |  |  |
| I have received enough advice from supervisors to assist in planning my career. |  |  |  |  |  |
| I often feel stressed. |  |  |  |  |  |
| The number of cases treated in this residency program is adequate. |  |  |  |  |  |
| Sometimes I feel like a failure. |  |  |  |  |  |
| I am often assigned to care for patients with whom I do not have enough experience to deal. |  |  |  |  |  |
| I often feel tired. |  |  |  |  |  |
| Sometimes I have emotional reactions that I feel bad about later. |  |  |  |  |  |
| I often feel overwhelmed. |  |  |  |  |  |
| The degree of responsibility I have for caring for patients is adequate. |  |  |  |  |  |
| I rarely have time to read. |  |  |  |  |  |
| The dedicated professors contribute in large part to the teachings I have received. |  |  |  |  |  |
| The average number of calls (requests for exams, beeps, urgencies, complications) on duty days is reasonable. |  |  |  |  |  |
| I generally feel that other residents are helpful and “do their part”. |  |  |  |  |  |
| I receive sufficient personal support from supervisors. |  |  |  |  |  |
| I often feel depressed. |  |  |  |  |  |
| The administrative support offered by the residency program is sufficient for the exercise of my function. |  |  |  |  |  |
| I receive enough instruction on what is expected of me at each stage of my training. |  |  |  |  |  |
| The amount of work on this program is usually excessive. |  |  |  |  |  |
| I think I'm getting easily annoyed. |  |  |  |  |  |

**GENERAL ANXIETY DISORDER-7 (GAD-7)**

Spitzer RL, Kroenke K, Williams JBW, Löwe B. A Brief Measure for Assessing Generalized Anxiety Disorder: The GAD-7. Arch Intern Med. 2006 May 22;166(10):1092.

Over the last 2 weeks, how often have you been bothered by any of the following problems?

0 - Never

1 - Several days

2 - More than half the days

3 - Almost every day

| **Questions** | **0** | **1** | **2** | **3** |
| --- | --- | --- | --- | --- |
| Feeling nervous, anxious or very tense |  |  |  |  |
| Not being able to prevent or control concerns |  |  |  |  |
| Worry a lot about different things |  |  |  |  |
| Difficulty to relax |  |  |  |  |
| Being so agitated that it is difficult to remain seated |  |  |  |  |
| Easily upset or irritated |  |  |  |  |
| Feeling afraid as if something horrible was going to happen |  |  |  |  |

**PATIENT-9 HEALTH QUESTIONNAIRE (PHQ-9)**

Kroenke K, Spitzer RL, Williams JBW. The PHQ-9: Validity of a brief depression severity measure. J Gen Intern Med. 2001 Sep;16(9):606–13.

Over the last 2 weeks, how often have you been bothered by any of the following problems?

0 - Never

1 - Several days

2 - More than half the days

3 - Almost every day

| Questions | **0** | **1** | **2** | **3** |
| --- | --- | --- | --- | --- |
| Little interest or little pleasure in doing things |  |  |  |  |
| Feeling “down”, depressed or hopeless |  |  |  |  |
| Trouble falling or staying asleep, or sleeping more than usual |  |  |  |  |
| Feeling tired or having little energy |  |  |  |  |
| Poor appetite or overeating |  |  |  |  |
| Feeling bad about yourself - or thinking that you are a failure or that you have disappointed your family or yourself |  |  |  |  |
| Trouble concentrating on things, such as reading the newspaper or watching television |  |  |  |  |
| Moving or speaking so slowly that other people could have noticed. Or the opposite - being so agitated or restless that you have been moving around a lot more than usual |  |  |  |  |
| Thoughts that you would be better off dead, or of hurting yourself |  |  |  |  |

**MULTIDIMENSIONAL SCALE OF SOCIAL EXPRESSION (MSSE-M)**

Caballo, Vicente E. La multidimensionalidad conductual de habilidades sociales: Propiedades psicométricas de una medida de autoinforme, la EMES-M. Psicologia Conductual. 1993;1(2):221–31.

The following questionnaire is designed to provide information about how you normally act. Please answer the questions by placing an X in the appropriate place (from 0 to 4), according to your own choice:

0 = Never or very rarely (0 to 9% of the time)

1 = Rarely (10% to 34% of the time)

2 = Once in a while (35% to 65% of the time)

3 = Normally or frequently (66% to 90% of the time)

4 = Always or very often (91% to 100% of the time)

| **Questions** | **0** | **1** | **2** | **3** | **4** |
| --- | --- | --- | --- | --- | --- |
| When people I barely know praise me, I try to minimize the situation, regardless of the fact for which I am praised. |  |  |  |  |  |
| When people pressure me to do things for them, I have a hard time saying "no". |  |  |  |  |  |
| I avoid asking questions of people I don't know. |  |  |  |  |  |
| I am unable to say no when my partner asks me for something. |  |  |  |  |  |
| When my superior or boss pisses me off, I am able to tell him that. |  |  |  |  |  |
| It is easy for me to make my partner feel good through compliments. |  |  |  |  |  |
| If I am in the cinema or in a lecture and there are two people speaking very loudly, I ask for silence. |  |  |  |  |  |
| When a person I find attractive asks me for something, I am unable to say "no". |  |  |  |  |  |
| When I get angry with someone, I don't show it. |  |  |  |  |  |
| I keep my opinions to myself. |  |  |  |  |  |
| If I am attracted to someone I don't know, I try hard to try to get to know her. |  |  |  |  |  |
| It is difficult for me to speak in public. |  |  |  |  |  |
| I am unable to demonstrate that I do not agree with my partner. |  |  |  |  |  |
| I avoid asking questions in class or at work out of fear or shyness. |  |  |  |  |  |
| It is easy for me to praise someone I know little. |  |  |  |  |  |
| It is difficult for me to make new friends. |  |  |  |  |  |
| If a friend breaks my trust, I clearly say that I am disappointed. |  |  |  |  |  |
| I express feelings of affection to my parents. |  |  |  |  |  |
| It is difficult for me to praise superiors. |  |  |  |  |  |
| If I were in a class or meeting and the teacher or the person who runs it made a statement that I disagree with, I would give my point of view. |  |  |  |  |  |
| If, in a restaurant, I am served food that is not to my taste, I complain about it to the waiter. |  |  |  |  |  |
| It is difficult for me to talk to someone that I find attractive and that I know little. |  |  |  |  |  |
| When I meet a person I like, I ask for his phone so we can meet again. |  |  |  |  |  |
| If I'm angry with my parents, I tell them that clearly. |  |  |  |  |  |
| I express my point of view even if others do not agree. |  |  |  |  |  |
| Starting a conversation with a stranger is difficult for me. |  |  |  |  |  |
| I am unable to defend my rights before my superiors. |  |  |  |  |  |
| If a superior criticizes me without reason, it is very difficult for me to discuss his criticism openly. |  |  |  |  |  |
| If a person I find attractive criticizes me unfairly, I clearly ask for explanations. |  |  |  |  |  |
| It is easy for me to approach and start a conversation with a superior. |  |  |  |  |  |
| When I meet new people, I can't talk much. |  |  |  |  |  |
| I pretend I didn't see it when someone ran out of line in front of me. |  |  |  |  |  |
| I am unable to tell someone I find attractive that I like her. |  |  |  |  |  |
| It is difficult for me to criticize others, even when I have reasons. |  |  |  |  |  |
| I don't know what to say to people I find attractive. |  |  |  |  |  |
| If I realize that I'm falling in love with someone I'm dating, I express my feelings to that person. |  |  |  |  |  |
| If a family member criticizes me unfairly, I easily express my anger. |  |  |  |  |  |
| It is easy for me to accept compliments from others. |  |  |  |  |  |
| When they complement me, I don't know what to say. |  |  |  |  |  |
| I am unable to speak in public. |  |  |  |  |  |
| I am unable to show affection to people I find attractive. |  |  |  |  |  |
| I avoid asking a person for something when it comes to a superior. |  |  |  |  |  |
| If a close and respected relative were bothering me, I would clearly express my annoyance. |  |  |  |  |  |
| If in a store the employee first attends someone who arrived after me, I call your attention. |  |  |  |  |  |
| It is difficult for me to give praise to someone I find attractive. |  |  |  |  |  |
| When I'm in a group, I have trouble finding things to talk about. |  |  |  |  |  |
| It is difficult for me to show affection to another person in public. |  |  |  |  |  |
| If a neighbor that I find attractive and that I would like to meet approached me when I left the house and asked the time, I would take the initiative to start a conversation. |  |  |  |  |  |
| I am a shy person. |  |  |  |  |  |
